# Mitochondrial replacement by genome transfer in human oocytes: Efficacy, concerns, and legality

**DOI:** 10.1002/rmb2.12356

**Published:** 2020-11-03

**Authors:** Mitsutoshi Yamada, Suguru Sato, Reina Ooka, Kazuhiro Akashi, Akihiro Nakamura, Kenji Miyado, Hidenori Akutsu, Mamoru Tanaka

**Affiliations:** ^1^ Department of Obstetrics and Gynecology Keio University School of Medicine Tokyo Japan; ^2^ Department of Reproductive Biology National Research Institute for Child Health and Development Tokyo Japan

**Keywords:** mitochondrial disease, mitochondrial DNA, mitochondrial DNA carryover, mitochondrial replacement, mtDNA genetic drift

## Abstract

**Background:**

Pathogenic mitochondrial (mt)DNA mutations, which often cause life‐threatening disorders, are maternally inherited via the cytoplasm of oocytes. Mitochondrial replacement therapy (MRT) is expected to prevent second‐generation transmission of mtDNA mutations. However, MRT may affect the function of respiratory chain complexes comprised of both nuclear and mitochondrial proteins.

**Methods:**

Based on the literature and current regulatory guidelines (especially in Japan), we analyzed and reviewed the recent developments in human models of MRT.

**Main findings:**

MRT does not compromise pre‐implantation development or stem cell isolation. Mitochondrial function in stem cells after MRT is also normal. Although mtDNA carryover is usually less than 0.5%, even low levels of heteroplasmy can affect the stability of the mtDNA genotype, and directional or stochastic mtDNA drift occurs in a subset of stem cell lines (mtDNA genetic drift). MRT could prevent serious genetic disorders from being passed on to the offspring. However, it should be noted that this technique currently poses significant risks for use in embryos designed for implantation.

**Conclusion:**

The maternal genome is fundamentally compatible with different mitochondrial genotypes, and vertical inheritance is not required for normal mitochondrial function. Unresolved questions regarding mtDNA genetic drift can be addressed by basic research using MRT.

## Graphical abstract

This review discusses current approaches and legality regarding mitochondrial replacement for preventing the inheritance of mitochondrial diseases such as encephalomyopathy, cardiomyopathy, hearing loss, diabetes, and renal impairment, caused by pathogenic mutations in mtDNA. Although relevant advances have been achieved in the field of treatments for preventing mutant mtDNA transmission, some concerns and challenges remain such as mtDNA carryover, mtDNA genetic drift, and donor‐recipient compatibility.

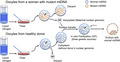

## 1 INTRODUCTION

Mitochondria are essential for the normal functions of multicellular life. Human mitochondria contain a residual genome (mitochondrial DNA, mtDNA), which is critical for the functions of mitochondria in energy production through oxidative phosphorylation (OXPHOS), calcium and reactive oxygen species (ROS) signaling, and regulation of apoptosis. mtDNA is a double‐stranded, circular molecule of 16 569 nucleotide pairs containing 37 genes encoding two rRNAs, 22 tRNAs, and 13 protein‐coding sequences, which are distributed among the OXPHOS protein complexes I, III, IV, and V, and are involved in OXPHOS activity. The displacement loop (D‐loop) is a regulatory sequence that controls mtDNA replication and transcription. In contrast, the nuclear genome encodes 74 polypeptides, including an OXPHOS complex and other mitochondrial proteins.

Mutations in mtDNA cause defects in the respiratory chain function and energy production within mitochondria, which can result in a wide range of clinical conditions and mitochondrial diseases. Thus far, there is no fundamental treatment for mitochondrial diseases. Thus, mitochondrial replacement (MR) for preventing the inheritance of mutant mtDNA has been tested in animal models, and clinical research is ongoing in the United Kingdom (UK). Here, we reviewed recent developments in human models of MRT, the underlying biology, and the regulatory guidelines for current methods, particularly in Japan.

## 2 mtDNA MUTATIONS AND MITOCHONDRIAL DISEASES

Many somatic cells contain approximately 1000 copies of mtDNA, whereas oocytes contain several hundred thousand copies of mtDNA.^1^ Dysfunction in mitochondrial replication can result in single or multiple mtDNA mutations. The mitochondrial genome mutation rate is reported to be between two‐ and sixfold higher in non‐vertebrates, and approximately 20 times higher in vertebrates, than that of nuclear genomes.^2^ To date, the mechanisms that affect variations in mtDNA mutation rates are not well understood, although several hypotheses have been suggested. First, the different metabolic rates of various species can lead to higher ROS production during OXPHOS in mitochondria, resulting in oxidative damage and higher mtDNA mutation rates. Second, the number of genome replications per generation differs between mitochondrial and nuclear genomes. Furthermore, DNA polymerase γ, which is involved in the replication of the mitochondrial genome, has poor fidelity.^2^

Mitochondrial disruption of energy production due to mtDNA mutations can affect different tissues and result in severe disease. Mutations in mtDNA can cause defects in the respiratory chain function and energy production within mitochondria, resulting in a wide range of clinical conditions, such as liver dysfunction, bone marrow failure, pancreatic islet cell deficiency, diabetes mellitus, deafness, respiratory failure, stroke, heart disease, neurodegenerative disorders, and other diseases, collectively referred to as mitochondrial diseases.^3^ Diseases resulting from mutant mtDNA show distinct patterns of inheritance owing to three features: maternal inheritance, mtDNA heteroplasmy (the proportion of mutant mtDNA relative to total mtDNA in a cell), and mtDNA replicative segregation.

The first feature refers to mutant mtDNA being subjected to maternal transmission through the oocyte cytoplasm and not from the father. However, some exceptions have been reported; for example, 17 individuals who inherited mtDNA from both parents were identified using sequencing data from the mitochondrial genomes of members from three unrelated families.^4^ The second type, mtDNA heteroplasmy, is wherein phenotypic variability of mtDNA diseases is related to the high copy number of mtDNA in mammalian cells, which can therefore contain both mutant and wild‐type mtDNA populations.^5^ The third type, mtDNA replicative segregation, can yield various distinct genotypes in the mtDNA pool of each tissue.^6^ Mitochondrial disease severity is dependent on the affected gene, percentage of mtDNA heteroplasmy and tissue distribution.

Two mitochondrial genetic bottlenecks have been reported to date. During oogenesis, primordial germ cells containing mutant mtDNA exhibit a dramatic reduction in mtDNA content, with discrete mitochondria containing approximately five mtDNA molecules.^7^ Oocytes receive only a selected number of mtDNA molecules each, which are amplified accordingly to yield several hundred thousand mtDNA copies, as is observed in mature oocytes.^8^ This selective reduction in mtDNA copies ensures that very few mtDNA copies can be clonally amplified, resulting in variability in the percentage of mutant mtDNA molecules in gametes and transmission to the next generation. This is referred to as the mitochondrial genetic bottleneck, and results in different loads of mutant mtDNA in different oocytes and variable transmission of pathogenic mtDNA from mother to offspring.

mtDNA mutations are found in approximately 1 of 200 live births.^9^ Human mitochondrial disease‐causing mtDNA mutations were originally reported in 1988.^5^, ^10^ Since then, over 200 such mtDNA mutations have been discovered (as shown in mitomap [https://www.mitomap.org/MITOMAP]); most of these mutations occur in a heteroplasmic context. Mitochondrial diseases affect approximately 1 out of 5000‐10 000 adults.^11^ However, owing to difficulties in diagnosis due to an extraordinarily broad spectrum of symptoms, the actual incidence is thought to be much higher. Variability in the degree of mtDNA heteroplasmy arising from the mtDNA bottleneck can affect the phenotypes of mitochondrial diseases. There is a phenotypic threshold effect associated with the percentage of mutant mtDNA per cell, and its severity varies greatly depending on the specific mtDNA mutation and the organ affected.^3^

## 3 PREVENTION OF THE TRANSMISSION OF ABNORMAL mtDNA USING PRE‐IMPLANTATION GENETIC TESTING FOR MITOCHONDRIAL DISORDERS

Currently, no curative treatments for mitochondrial diseases have been developed, and treatment remains limited to symptomatic management. Increasing identification of mutations and advanced genomic techniques have improved the diagnosis of mitochondrial disorders. In clinical practice, pre‐implantation genetic testing (PGT), an in vitro fertilization (IVF)‐based technique developed three decades ago,^12^ is one option to produce embryos without mutant mtDNA or with few heteroplasmic mtDNA mutations. PGT‐M is the approach used for the diagnosis of mitochondrial disease, and PGT is conventionally used for monogenic disorders. In the current review, the term PGT‐MIT would be used for convenience to describe PGT, which is limited to the diagnosis of mitochondrial diseases. PGT is based on the genetic analysis of one or several cells obtained from embryos at the cleavage stage^13^ and/or the blastocyst stage.^14^, ^15^, ^16^, ^17^ This approach enables the selection of mutation‐free embryos or embryos with low mutation load, before their transfer to the uterus. Since the first successful case in which PGT‐MIT reduced the risk of neurogenic ataxia retinitis pigmentosa in a patient with the m.8993T > G mutation,^18^ a number of children have been born with the help of PGT‐MIT, by enabling the selection of embryos with low mutation levels.^19^, ^20^, ^21^

Although current conventional approaches are useful for reducing or eliminating the risk of mtDNA, pre‐implantation genetic diagnosis has several limitations. For example, pre‐implantation genetic diagnosis is not available for women who harbor homoplastic mtDNA mutations. Additionally, animal studies using experimentally constructed primate embryos containing heteroplasmic mtDNA showed marked variation in the levels of heteroplasmic mtDNA between blastomeres,^22^ suggesting the possibility of misdiagnosis of mitochondrial disease. Although strong correlations in mtDNA heteroplasmy levels between the trophectoderm and the remaining blastocyst have been reported in human embryos,^20^, ^22^ few studies have reported low levels of heteroplasmic variation between blastomeres of cleavage‐stage embryos.^19^, ^20^, ^21^ Embryo biopsy may have detrimental effects on development because PGT for chromosome aneuploidy (PGT‐A) was shown not to improve the overall pregnancy outcomes.^23^ However, initial studies have shown the presence of DNA in polar bodies,^24^ blastocoel fluid, and spent culture medium,^25^ which could facilitate the development of non‐invasive methods for PGT.

## 4 MR THERAPY (MRT)

The severity of clinical phenotypes in most maternally inherited mitochondrial diseases varies according to the percentage of mutant mtDNA (mtDNA heteroplasmy). Although PGT is a useful option for reducing the risk of abnormal mtDNA transmission, the exact threshold for the mtDNA heteroplasmy ratio is still unclear.^26^ Even if embryos containing a mutation ratio below the threshold give rise to a successful pregnancy, the stochastic replicative segregation of heteroplasmic mtDNA during cell division can result in genetic mosaicism within each organ. Therefore, to reduce the risk of mtDNA disease transmission, approaches to replace abnormal mitochondria with normal mitochondria from healthy donors using the nuclear transfer (NT) technique have been proposed.

**Figure 1:**
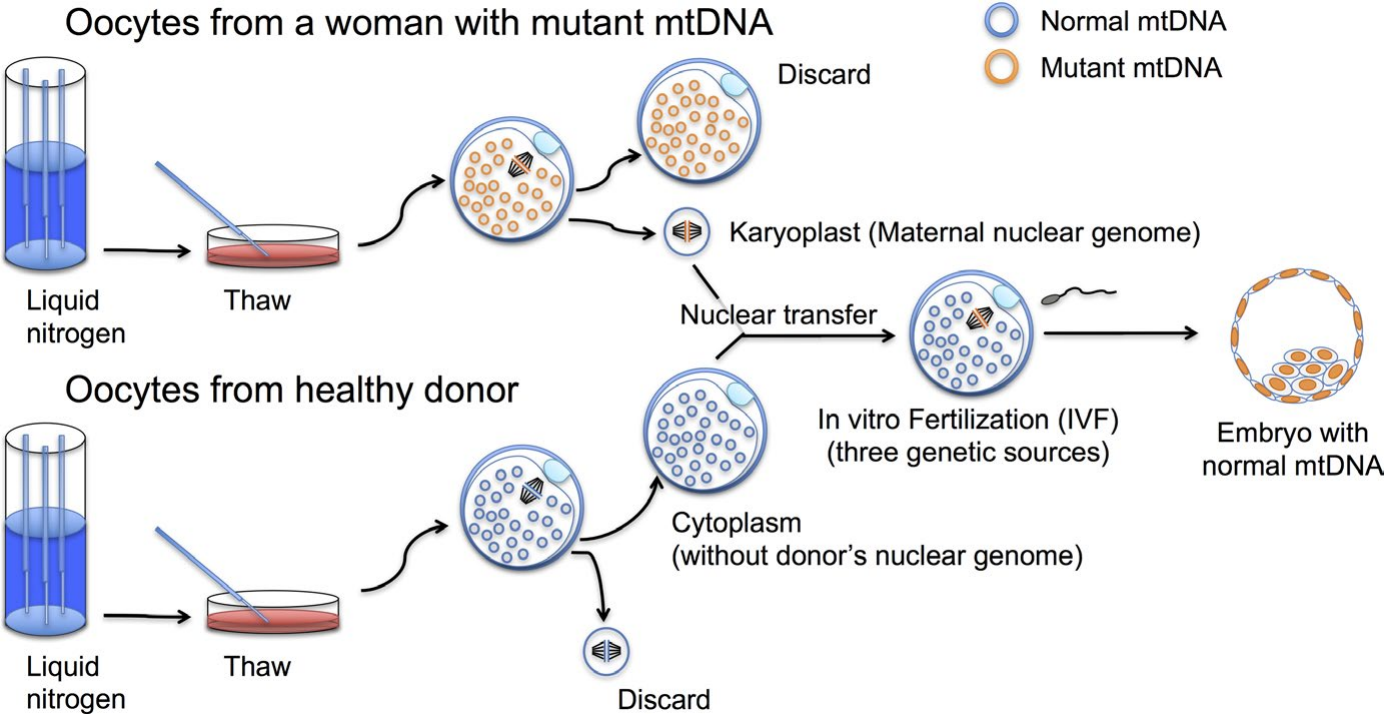
Spindle transfer for mitochondria replacement. Spindles are enucleated from both oocytes, and the carrier's spindle (orange) is fused with the enucleated healthy donor oocyte (blue). After nuclear exchange, the resulting oocyte, which consists of nuclear DNA from the carrier and cytoplasm from the donor oocyte, is subjected to in vitro fertilization (IVF) with the partner's sperm

The nuclear genome can be transferred by NT techniques, such as maternal spindle transfer (MST; Figure 1) and germinal vesicle transfer from unfertilized oocytes or first polar body transfer (PB1T), second polar body transfer (PB2T), and pronuclear transfer (PNT) from zygotes (Table 1). MST is the procedure for transferring the karyoplast (which contains nuclear DNA with a small amount of cytoplasm, surrounded by the plasma membrane) into an enucleated donor oocyte before fertilization. Because these reconstructed oocytes or embryos are obtained from healthy donor oocytes or zygotes, such NT procedures are thought to reduce the risk of transmission of mutant mtDNA to the next generation. Several studies have reported the live birth of healthy offspring from mouse embryos at the pronucleus stage^27^ and from rhesus macaque oocytes at the metaphase stage of meiosis II (MII).^28^

**Table 1:** Mitochondrial DNA (mtDNA) carryover after genome transfer

	Maternal Spindle Transfer (MST)	First Polar Body Transfer (PB1T)	Second Polar Body Transfer (PB2T)	Pronuclear Transfer (PNT)
Genetic material of recipient	MII spindle	first polar body	second polar body	pronuclei
Genetic material of donor	cytoplasm of MII oocyte	cytoplasm of MII oocyte	cytoplasm of embryo at the pronucleus stage	cytoplasm of embryo at the pronucleus stage
mtDNA copy number in karyoplast	2318^38^ (mouse); 1129 ± 785^1^ (human)	359^38^ (mouse)	1092^38^ (mouse)	34 392^38^ (mouse)
mtDNA carryover in oocytes/1‐cell embryo	0.36% (total mtDNA within MII oocytes 311 146 ± 206 521),^1^ 0.5%,^32^ 0.2%‐0.8%,^42^ 1‐cell embryo 1.03%^31^ (human)	0.38%^31^ (human)	N/A	8.1 ± 7.6%^29^ (human)
mtDNA heteroplasmy in pre‐implantation embryo	cleavage stage 0.27%,^1^ 0.36%‐0.47%,^31^ morula and blastocyst stages 0.39%,^1^ 0.36%‐0.69%,^31^ 0%‐0.9%,^32^ 0.4%‐0.9%^42^ (human)	N/A	N/A	1.68 ± 1.81%,^29^ <2% in the majority of PNT blastocysts, none with > 5%^35^ (human)
mtDNA heteroplasmy in stem cells	0.00%‐2.79%,^1^ 0.00%‐1.70%,^32^ 0.0%‐53.2% at passage 36,^31^ ≤1%‐100% ^42^ (human)	N/A	N/A	0.0‐ <~80%^35^ (human)
mtDNA heteroplasmy in offspring	5%‐44%,^27^ 5.5 ± 1.4%^38^ (mouse); 2.36%‐9.23% (amnion 6.77%, buccal epithelium 3.52%, foreskin 9.23%, hair follicles 5.59%, urine precipitate 2.36%)^44^ (human)	0%^38^ (mouse)	1.7 ± 2.8%^38^ (mouse)	23.7 ± 11.1%^38^ (mouse)

Note

The references in the table are shown as numbers in square brackets.

In an early human study, Craven et al reported a successful rate of blastocyst development after the transfer of pronuclei obtained from abnormal human embryos (unipronuclear or tripronuclear).^29^ Their findings showed a decrease of approximately 50% in the blastocyst formation rate after genome exchange (8.3%, 3/36) compared with that of unmanipulated embryos (n = 76), partly owing to the absence of either a maternal or paternal genome. There was variation in mtDNA carryover, that is, the transfer of the mtDNA genotype from the nucleus donor to the embryos receiving pronuclei (8.1 ± 7.6%, n = 8). With optimization of the NT technique, the average mtDNA heteroplasmy ratio at pre‐implantation stages decreased by less than 2% (1.68 ± 1.81%, n = 9).

The Nuffield Council on Bioethics published a report in 2012 titled “Novel techniques for the prevention of mitochondrial DNA disorders: an ethical review.” In this report, the authors detailed their approval for research on the prevention of mtDNA disorders.^30^ Paull et al performed genome exchange at the MII stage (MST)^1^ and used artificial activation of unfertilized oocytes to exclude the influence of paternal factors on nuclear DNA‐mtDNA incompatibility. The blastocyst development ratio in genome‐exchanged oocytes (37%, 7/18) was comparable to those in unmanipulated IVF embryos (52%, 54/103) and parthenotes (33%, 7/21). In addition, the successful ratio of stem cell derivation from genome‐exchanged oocytes (15%, 3/18) was also comparable to that from parthenotes (19%, 4/21). To estimate the amount of potential mtDNA carryover, mtDNA copy number was quantified by quantitative polymerase chain reaction. The results showed that karyoplasts had an average mtDNA copy number of 1129 ± 785 (n = 22) and total mtDNA in MII oocytes of 311 146 ± 206 521 (n = 5; estimated 0.36% mtDNA carryover). The mtDNA heteroplasmy ratios in pre‐implantation stages (cleavage stage: 0.27%, n = 17; morula and blastocyst stages: 0.39%, n = 7) and stem cells remained low (undetectable in stem cell lines; 2.79 ± 0.27% in one stem cell line at passage 4 [p4]–p10, becoming undetectable at p14). Nuclear genome transfer did not reduce developmental efficiency of the blastocyst stage, as demonstrated by parthenogenetically activated oocytes after genome transfer.^1^, ^31^ Tachibana et al performed MST, and reconstructed oocytes were fertilized by intracytoplasmic injection. Although almost half of the genome‐exchanged zygotes showed an abnormal fertilization pattern (1PN or multiple pronuclei 52%, 23/44), the blastocyst developmental rate was comparable to that of unmanipulated embryos, and isolated stem cells were karyotypically normal.^32^

## 5 MINIMIZING mtDNA CARRYOVER

To avoid mtDNA heteroplasmy in MRT, it is critical to ensure inheritance of a single maternal mtDNA lineage. The human egg contains more than 100 000 copies of mtDNA; however, the sperm contains only approximately 100 copies.^33^ Although a few reports have shown that paternal mtDNA can be passed on to the offspring,^4^ sperm mitochondria are ubiquitinated inside the oocyte cytoplasm and later subjected to proteolysis during pre‐implantation development.^34^ Unlike after natural fertilization, when ubiquitination and proteolysis can eliminate a small amount of paternal mtDNA heteroplasmy, technical improvements are needed to avoid mtDNA heteroplasmy after MRT.

Hyslop et al modified the PNT protocol so that pre‐implantation embryo development could be improved by minimizing mtDNA carryover.^35^ In the original protocol, sucrose was added to the manipulation medium to facilitate enucleation and fusion. However, it was later removed because of the osmotic effect, which may increase mtDNA carryover. Before karyoplast fusion, the enucleated karyoplast was vitrified. Excess cytoplasm from karyoplasts was removed before fusion, and karyoplasts were then fused with enucleated fresh cytoplasm; 79% of blastocysts showed less than 2% mtDNA heteroplasmy, and none showed greater than 5% mtDNA heteroplasmy.

Polar body‐based nuclear substitution may be useful for minimizing mtDNA carryover after genome transfer. The first polar body contains a subset of bivalent chromosomes, whereas the second polar body contains a haploid set of chromatids. A mouse study showed that polar bodies could contribute to efficient full embryo development.^36^, ^37^ Additionally, Wang et al studied mtDNA carryover after genome transfer between the spindle (MST), pronuclear (PNT), first polar body (PB1T), and second polar body (PB2T) in a mouse model.^38^ PBT prevented the transmission of mtDNA variants, and the reconstructed embryos supported normal fertilization and produced live offspring. Strikingly, genetic analysis confirmed that PBT offspring (F1) possessed minimal donor mtDNA carryover (F1‐PB1T: 0%; F1‐PB2T: 1.7%) compared with the spindle (F1‐ST: 5.5%) and low pronuclear (F1‐PNT: 23.7%) transfer, with a low mtDNA heteroplasmy ratio compared to the next generation (F2) following natural mating (F2‐PB1T: 0%, F2‐PB2T: 2.9%, F2‐ST: 7.1%, F2‐PNT: 22.1%). Additionally, PBT extends donor genome sources and increases efficiency when performed together with oocyte chromosome transfer. In human studies, we showed that mtDNA carryover after PBT was low (0.38%, n = 3).^31^ Moreover, Zhang et al showed that PB1T (transfer of cryopreserved first polar bodies into fresh cytoplasm) did not compromise fertilization (75.0%) or blastocyst formation rates (50.0%) after PB1T, when compared with unmanipulated control oocytes (84.2% and 43.8%, respectively). Ma et al also showed that although pre‐implantation development after PB1T was compromised (42%) compared with that of unmanipulated embryos (75%), isolated embryonic stem cell (ESC) lines from PB1T‐reconstructed embryos showed similar DNA methylation and transcriptome profiles. These results suggest that the PBT technique may enable efficient birth of live offspring with minimal mtDNA carryover and could be a therapeutic option for mitochondrial disease.

## 6 MITOCHONDRIAL GENETIC DRIFT (mtDNA GENOTYPE REVERSION)

The mechanism of mtDNA replicative segregation is still not clear. Even with minimal mtDNA carryover, there is concern that mitochondrial haplotypes may affect the predominance of one haplotype over another, resulting in mtDNA genotype reversion. Therefore, genome transfer between oocytes of women with different mitochondrial haplotypes was performed. Twelve ESC lines were isolated from parthenogenetically activated mitochondria replacement oocytes. Paull et al showed that the amount of mtDNA contained in the karyoplast generally accounts for less than 2% of MR embryos. Four stem cell lines isolated from MRT embryos showed complete loss of mtDNA heteroplasmy.^1^ Yamada et al isolated eight additional stem cell lines. No mtDNA carryover (heteroplasmy) was detected in four of the eight cell lines, whereas the other four contained 0.2% to 1.7% mitochondrial heteroplasmy at derivation (average: 0.42%).^31^ For all but one cell line, mitochondrial heteroplasmy decreased below the limit of detection by passage six and remained stable for more than 30 passages or more than 6 months of culture (haplotype cytoplasm donor: nucleus donor HV:I, A:L3, L0:L3, L3:U, and L3:HV). In contrast, although there were small amounts of mtDNA carried over during genome transfer for MR, one of the eight stem cell lines (H1:L3) showed that the mtDNA heteroplasmy of the H1 haplotype, which was carried from the nuclear donor, increased from 1.3% at derivation to 53.2% at passage 36.

Somatic cell nuclear transfer (SCNT) introduces a small amount of mtDNA copies of somatic cells (thousands of copies) into oocytes (several hundred thousand copies of a different mtDNA genotype).^1^ Therefore, SCNT can be a good model for observing mitochondrial dynamics after genome transfer. Accordingly, we analyzed mtDNA heteroplasmy in 12 NT‐ESC lines.^39^, ^40^ Two of the 12 NT‐ESC lines showed a directional mtDNA drift toward the K1 haplotype (somatic mtDNA genotype) and decrease in haplotype L0 (oocyte mtDNA genotype) between passages 0 and 10, reaching homoplasmy of the somatic mtDNA genotype between passages 15 and 25. However, a third NT‐ESC line from the same haplotype combination contained no detectable K1 mtDNA at the point of derivation and remained homoplasmic for the L0 haplotype for over 40 passages. Overall, among 24 cell lines with 14 different combinations of mtDNA haplotypes, three cell lines of two different combinations (K1 with L0 and H1 with L3) showed mtDNA genotype instability.

Similarly, mtDNA genotype instability during cell differentiation into fibroblasts or cardiomyocytes was also observed. Furthermore, Lee et al showed mtDNA genetic drift during monkey embryonic development.^22^ Sharpley et al used heteroplasmic mice with roughly equal proportions of 129S6 and NZB mtDNAs, generated by fusion of NZB mtDNA‐containing cytoplasts to 129S6‐derived female embryonic stem cells and transfer of heteroplasmic stem cells into blastocysts. Mitochondrial heteroplasmy led to mitochondrial genome instability and altered the composition of the two normal mitochondrial genotypes in differentiated tissues. NZB‐129 heteroplasmic mice had reduced activity, food intake, respiratory exchange ratio, accentuated stress response, and cognitive impairment compared with homoplasmic mice.^41^

Consistent with these findings, Hyslop et al observed a progressive increase in mtDNA carryover from ~4% to 60% in one out of five ESC lines derived from PNT blastocysts, regardless of the NT technique.^35^ Kang et al observed a similar phenomenon in three out of 18 ESC lines derived from blastocysts that were developed from reconstructed swapped oocytes with cytoplasm from healthy donors and spindles from patients harboring a pathogenic mtDNA mutation m.3243A → G, the cause of Leigh syndrome.^42^

To elucidate the mechanism of mtDNA genetic drift, we hypothesized that specific mitochondrial nuclear genotype combinations may confer cellular survival and/or proliferative advantages because of differences in mitochondrial function. Accordingly, the mtDNA heteroplasmy ratio in mixed cells whose mtDNA genotypes were homoplasmic for haplotype L0 or haplotype K1 and mixed at different ratios was observed over eight passages or 6 weeks. The composition of cultures remained stable, and the proportion of cells with haplotype K1 did not increase, suggesting that haplotype K1 did not confer a survival or proliferation advantage compared with cells with haplotype L0.^31^

Kang et al proposed that a polymorphism within the conserved sequence box II (CSBII) region of the D‐loop identified in the mtDNA of nuclear donors may have a conferred replicative advantage, which could be helpful for determining the eligibility of donor oocytes. Although two of the three stem cell lines that harbor CSBII haplotypes G6AG8:G5AG8 reverted, one of them did not revert.^42^ Similarly, ESC lines harboring CSBII haplotypes G6AG7:G6AG7 and G6AG8:G6AG7 showed mtDNA instability,^43^ suggesting that mtDNA variants within the D‐loop region itself cannot explain the cause of mtDNA reversion. Although mtDNA genetic drift could be a stem cell‐specific phenomenon that is not observed during in vivo human development, this possibility should be considered in clinical applications.

## 7 ETHICAL CONCERNS AND LEGAL CONTROVERSIES REGARDING DELIVERY AFTER GENOME TRANSFER FOR THE PREVENTION OF MITOCHONDRIAL DISEASE

Despite ethical concerns and legal controversies, the New York City Clinic (USA) reported the first live human birth after genome transfer between a patient with mitochondrial disease (Leigh syndrome) and a healthy donor in 2017. Following the use of MST to reduce transmission of the m.8993T > G mutation, which causes Leigh syndrome, a male euploid blastocyst containing a 5.7% mtDNA mutation load was transferred *in utero*. Subsequently, the neonate was born with an mtDNA mutation load between 2.36% and 9.23% in each tissue (amnion, 6.77%; buccal epithelium, 3.52%; foreskin, 9.23%; hair follicles, 5.59%; and urine precipitate, 2.36%). The baby was reported to be healthy at 7 months of age.^44^ Another baby was born in Ukraine using the MRT technique.^45^ In this case, genome transfer was not used for disease prevention, but instead for rejuvenation of aging oocytes to treat embryo arrest.

In the United States of America, including in New York state, clinical research on MST is prohibited (Public Law No. 115‐31 §736, 2017), and research funding is also prohibited. The procedure was carried out in Mexico, where there is no legal regulation. However, one report of a study in mice showed that mixing differently derived mitochondria can lead to neurological and metabolic diseases.^26^ Long‐term follow‐up of the child is important so that we can validate the efficacy and safety of MRT. Additionally, it is essential that the physicians in charge of the study provide sufficient information, including potential risks, to the patients prior to the study, so that clients may properly understand the efficacy, risks, and importance of long‐term follow‐up, which would benefit both the child and future research on mitochondrial diseases. To date, it is still illegal to transfer embryos that have undergone germ line changes, including MRT embryos, in the United States of America. The US Food and Drug Administration is not currently hearing arguments for the use of any germ line changes in gametes.

In the UK, after being debated by the Human Fertilization and Embryology Authority and both Houses of Parliament in 2015, the Mitochondrial Donation Regulations passed the MRT into law. Subsequently, in December 2016, MRT was approved for clinical use in a limited number of cases, and further institutional accreditation was granted to the Newcastle Fertility Center in March 2017. A governing body determines whether a patient is suitable for treatment based on an individual petition for each case, and only heritable known diseases caused by mutations are considered. However, the following requirements are advocated strictly: highly skilled embryologists to perform the technique, the need for pathways to ensure appropriate genetic counseling for women with mitochondrial diseases and egg donors, and further long‐term follow‐up of children born as a result of MRT.^46^, ^47^ Notably, the use of MRT to treat infertility is unlawful.

## 8 REGULATING THE HANDLING OF NINE TYPES OF EMBRYOS PRODUCED BY CLONING TECHNIQUES AS “SPECIFIED EMBRYOS” IN JAPAN

In terms of policy responses to ethical issues in medical and life science research in Japan, from the perspective of respect for academic freedom as stipulated in Article 23 of the Japanese Constitution, consideration for self‐regulation by expert groups, and the ability to flexibly respond to changes in social conditions and technological progress in a timely manner, regulations with penalties and laws have been strictly limited.

In Japan, the first IVF infant was born in 1983; however, no laws or regulations have been established regarding assisted reproductive health, similar to those in the UK. With regard to the use of human embryos in research, the Japanese Society of Obstetrics and Gynecology has autonomously regulated the members of the society according to the guidelines outlined in the publication “Notice on research dealing with human sperm, eggs, and fertilized eggs (1985)” and research on the improvement of assisted reproductive technology has been extensive. However, the announcement of the birth of the somatic cell cloned sheep Dolly in 1997 raised international concerns regarding the application of cloning technology to humans. The Bioethics Committee of the Science and Technology Council of Japan established a subcommittee on cloning in 1998 to carry out specialized studies.

**Figure 2:**
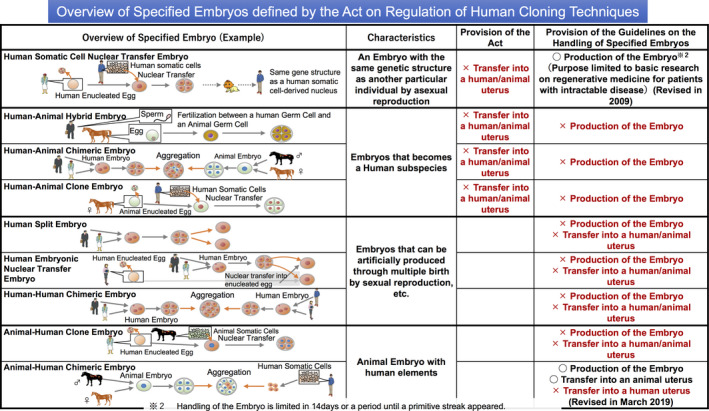
Overview of specified embryos defined by the Act on Regulation of Human Cloning Techniques. Embryos produced by genome transfer techniques are categorized as “Specified Embryos” in the Act on Regulation of Human Cloning Techniques. No person shall transfer a human somatic cell nuclear transfer (SCNT) embryo, human‐animal hybrid embryo, human‐animal clone embryo, or human‐animal chimeric embryo into a human or animal uterus. The image was adapted from the Announcement of Ministry of Education, Culture, Sports, Science and Technology‐Japan^50^

Cloning technologies are different from human embryo research for assisted reproductive technology. Because the birth of cloned humans or human‐animal hybrids may seriously affect human dignity, the Act on Regulation of Human Cloning Techniques (Act No. 146 of 2000) was enacted in 2000 to regulate cloning technology. Although several news and review articles have indicated that Japan had previously released draft guidelines with a more permissive stance on human embryo genome editing, which did not outlaw germline editing for reproduction,^48^, ^49^ anything that is intended for reproduction, including human cloning or hybrid individuals, is strictly prohibited. Despite significant changes in society and social values, laws cannot be quickly amended to respond to them; therefore, in Japan, research using germline cells has been carried out through the flexible application of guidelines, academic opinions, and bulletins. In 2001, the “Guidelines for the Handling of Specified Embryos” were enacted based on the same law (announced by the Ministry of Education, Culture, Sports, Science and Technology in December 2001), and these guidelines were then enforced. The Cloning Technology Regulation Act classifies embryos created by cloning and similar techniques into nine specified embryos (Figure 2).^50^ Since PNT, an MR method, is the same technology used to clone fertilized eggs, it is defined as a human embryonic NT embryo, and the specified guidelines prohibit the creation and *in utero* transfer of such embryos.

Research to create specified embryos is an exception when there are a scientific rationale and social relevance, even among cloning techniques. Because basic research to create human embryo nucleus transplants for the purpose of elucidating the pathogenesis of mitochondrial diseases is not in direct conflict with the objectives of the cloning technology regulation law, it is necessary to revise the “Guidelines for the Handling of Specified Embryos” to allow the creation of human embryo nucleus transplants, while at the same time providing appropriate measures, such as prohibiting the transfer of nuclear transplant‐generated human embryos into human or animal wombs. In Japan, the direction of human embryo research has been decided on the basis of discussions by national experts. Specifically, the Council for Science, Technology and Innovation, Cabinet Office (CSTI; consisting of experts in medicine, biology, jurisprudence, ethics, and the general public) compiled a document titled “Basic Approaches to the Handling of Human Embryos” (hereinafter referred to as “the Basic Principle”) in 2004. This document highlights the following two basic principles for human embryo handling: i) human embryos should be handled as “emerging of potential human life” and ii) the destruction of human embryos is not allowed. Thus, the creation of new human fertilized embryos for use as research materials and handling of human fertilized embryos in a manner that may damage the embryos is prohibited. However, exceptions are allowed when the three requirements of scientific rationality, safety, and social relevance are met; the aforementioned human cloned embryos and animal assembly embryos are also accepted as meeting these requirements. According to the Basic Principle, the specified embryos should also be examined based on the principles of human fertilized embryos, as the medical utility of human embryonic genome transfer embryos for mitochondrial diseases has been established. However, further studies are required to determine how to treat these embryos.

In October 2015, the UK approved the clinical use of nuclear‐replaced fertilized embryos and inter‐egg nucleus replacement for the prevention of mitochondrial diseases. Accordingly, the Expert Committee on Bioethics examined the scientific rationale and social validity of genome editing technology in June 2016, in parallel with an examination of genome editing technology. The expert panel on bioethics published a supplementary report, titled “Basic Principles on Handling of Human Embryos” (2018), which suggests the need for guideline development to allow the use of nuclear replacement technology for basic research, such as research on mitochondrial disease using surplus human fertilized embryos. In contrast, the use of newly created embryos for mitochondrial disease research (including cases in which the gametes are fertilized using nuclear replacement technology) and that of human unfertilized oocytes for research purposes are prohibited. In 2019, the CSTI published the “Second Report on the revision of the Basic Principles on the Handling of Human Embryos” (hereinafter referred to as the “Second Report”). The Second Report urged the development of guidelines for basic research on hereditary/congenial diseases using gene editing technology, basic research using newly produced embryos for assisted reproductive technology with gene editing technology, research on function and dynamics of mitochondria in oocytes and fertilized embryos before and after fertilization, and the use of nuclear replacement technology in human fertilized embryos. In response to the decision by CSTI, the Ministry of Education, Culture, Sports, Science, and Technology is now expected to revise the Specified Embryo Guidelines to establish requirements for conducting proper research using human nuclear embryos. Although spindle transfer is still prohibited when accompanied by embryo creation, CSTI and the Expert Committee on Bioethics will continue to discuss the acceptability of research using spindle transfer and ethical issues related to egg donation for research, through discussions with medical professionals, scientists, and mitochondrial patient groups.

## 9 CONCLUSION

To date, a few studies have described uncommon mtDNA mutations, and it is important to understand the unique genetic patterns and different behaviors of each mutation and to sufficiently explain uncertainties regarding the roles of these mutations in diseases during genetic counseling. There is increasing evidence supporting MR therapy by genome transfer technology to prevent the transmission of mitochondrial disease. Depending on the results of clinical trials in the UK, the research environment for MRTs could change substantially in the near future. Thus, PGT‐MIT is still an important process. Data from animal models of mitochondrial DNA disorders are not always applicable to humans.^51^ Public understanding of research using human fertilized eggs is essential to elucidate the lack of fundamental treatment for mitochondrial diseases and the need to develop innovative treatments. For safe and appropriate clinical application of MRT, nuclear transplant techniques need to be improved to reduce the amount of mtDNA carryover. Elucidation of the mechanisms of mtDNA genetic drift and the selection of compatible donors that do not compete with the recipient's mtDNA and nuclear DNA would also lead to better understanding of the etiology of mtDNA‐related diseases.

## DISCLOSURES

*Conflict of interest:* The authors declare that they have no conflicts of interest. *Human/animal rights:* This article does not contain any studies with human and animal participants performed by any of the authors.
